# Custom-Made 3D-Printed Titanium Implants for Managing Segmental Distal Tibial Bone Defects: A Systematic Literature Review

**DOI:** 10.3390/jcm14061796

**Published:** 2025-03-07

**Authors:** Viktor Dietrich Schick, Biagio Zampogna, Giovanni Marrara, Lorenza Siracusano, Leone Larizza, Salvatore Calaciura, Ilaria Sanzarello, Andrea Marinozzi, Danilo Leonetti

**Affiliations:** 1 BIOMORF Department of Biomedical and Dental Sciences and Morphological and Functional Imaging, Section of Orthopaedic and Trauma Surgery, University of Messina, A.O.U. Policlinico “G. Martino”—Via Consolare Valeria 1, 98124 Messina, Italy; viktor.schick@studenti.unime.it (V.D.S.); b.zampogna@policlinicocampus.it (B.Z.); giovanni.marrara@studenti.unime.it (G.M.); lorenza.siracusano@studenti.unime.it (L.S.); leone.larizza@unime.it (L.L.); salvatore.calaciura@studenti.unime.it (S.C.); ilaria.sanzarello@unime.it (I.S.); 2 Operative Research Unit of Orthopaedic and Trauma Surgery, Fondazione Policlinico Universitario Campus Bio-Medico, Via Alvaro del Portillo 200, 00128 Rome, Italy; a.marinozzi@policlinicocampus.it; 3 Research Unit of Orthopaedic and Trauma Surgery, Department of Medicine and Surgery, Università Campus Bio-Medico Di Roma, Via Alvaro del Portillo 21, 00128 Rome, Italy

**Keywords:** tibial segmental defect, non-union, custom-made implants, 3D-printed titanium implants

## Abstract

**Background:** The management of diaphyseal and distal tibial defects and non-unions is a significant challenge. Traditional treatments, such as distraction osteogenesis or Masquelet, are characterized by extended treatment times and elevated complication rates. Innovative approaches, such as customized 3D-printed titanium implants, are often required to restore structural integrity and function. This systematic review aimed to analyze the results achieved to date with this technique. **Methods:** A systematic review of the literature written in English was performed in PubMed, Scopus, and Cochrane to identify all cases of tibial non-unions or defects treated with customized 3D-printed titanium implants, excluding defects from tumor resection. Studies with a minimum of 12 months of follow-up were included. **Results:** The causes of treatment were infection in 10 patients, non-union in 6 patients, and severe bone loss after trauma in 3 cases. The size of the defect ranged from 3 to 8.5 cm. Osteointegration was 100% in all studies. The mean time to union was 5.3 months. The complication rate was 16%. **Conclusions:** Good results were reported in most patients. However, the data are insufficient to define the role of customized 3D-printed implants compared to traditional techniques. Further studies comparing them are needed to draw explicit guidelines.

## 1. Introduction

There is no universal definition of bone defects. A critically sized defect (CSD) generally refers to a bone loss that would not heal spontaneously and requires further specialized surgical intervention. The accurate size or volume of a bone defect for it to be considered “critically sized” is not well defined as it depends on the anatomic location and the condition of the soft tissues surrounding it. CSDs have multiple etiologies and can result from high-energy trauma, blast injury, infection, or tumor resection [1]. Non-union, on the other hand, is characterized by the failure of a fracture to consolidate approximately 6–9 months after the traumatic event [1,2]. Non-union is more frequent in areas where bone vascularization is scarcer or if the trauma is such as to compromise the vascularization of the fragments. In particular, open fractures, which are common in the tibia due to its subcutaneous location, often result in the loss of the initial fracture hematoma, periosteal stripping, and ischemic bone and soft tissues, increasing the risk of non-union [3]. Other risk factors associated with non-union include open reduction in a fracture, infection, post-surgical fracture gaps, wedges, or comminuted fractures, the degree of initial fracture displacement, and implants that do not provide adequate stability [4]. Furthermore, patient factors, such as advanced age, usage of non-steroidal anti-inflammatory drugs (NSAIDs), and smoking, may contribute to impaired bone healing [5].

Managing segmental tibial bone defects and non-unions presents significant challenges in orthopedic surgery, often necessitating innovative approaches to restore structural integrity and function. Traditional methods include autologous bone graft (ABG), vascularized fibular graft (VFG), distraction osteogenesis (DO, Ilizarov), or the induced membrane technique (IMT, Masquelet) [6].

ABG, harvested from the iliac crest or with the reamer–irrigator–aspirator (RIA) system, is the gold standard for treating bone defects less than 5 cm in size but is unsuitable for larger defects due to problems with graft resorption [7]. VFG is a surgical technique where a section of the fibula is transplanted with its intact blood supply (vascular pedicle) to repair the bone defect. While this technique allows larger defects to be filled, it is technically demanding as it requires microsurgical skills to reconnect the blood vessels from the donor site to the graft site. In addition to the technical difficulty, VFGs require a prolonged period of restricted weight bearing to allow for graft hypertrophy, and even then, up to 20% fracture in the first year [6]. Another drawback are potential complications at the donor site, including pain, muscle weakness, and sensory abnormalities. For these reasons, DO and the IMT are currently the preferred treatment options for segmental tibial defects [8]. Distraction osteogenesis, originally described by Ilizarov, makes use of an external fixator or an intramedullary nail to gradually transport an osteotomized segment of the bone to close the defect. It consists of three main phases: a latency phase that allows for an early reparative callus to form, an active distraction phase in which the bone segments are slowly pulled apart, stimulating the growth of new bone tissue to close the gap, and a consolidation phase in which the newly formed bone matures [6]. The technique requires a long external fixation period and a high level of compliance by patients, and up to 60% of cases are complicated by pin-tract infections [9,10]. The induced membrane technique, developed by Masquelet in the 1980’ is a two-stage surgical technique for the treatment of large bone defects. During the first stage, a temporary spacer made of antibiotic-loaded polymethylmethacrylate (PMMA) is inserted into the defect. This spacer induces the formation of a biologically active membrane, which hosts osteoinductive growth factors to aid in bone healing. During the second stage, typically 6–8 weeks later, the spacer is removed, and the resulting cavity is filled with autologous cancellous bone graft [11]. In contrast to DO, the external fixation time is reduced with the IMT because the external fixator can be converted to a plate or nail after the first stage has been completed. The IMT requires at least two invasive surgical procedures and infection rates of up to 43% have been reported [12].

Advancements in three-dimensional (3D) printing technology have paved the way for custom-made titanium implants, which offer a promising alternative for addressing these critically sized defects (CSDs) and non-unions, especially when other techniques fail. Three-dimensional printing technology enables the precise manufacture of implants tailored to each patient’s anatomical and pathological features. Like bone grafting techniques, 3D-printed implants allow for the immediate restoration of bone continuity because the implant fills the defect in a single step [4]. In addition, as with conventional methods, these implants can be used in conjunction with external or internal fixation techniques to facilitate early weight-bearing, thereby restoring the affected leg’s functionality [13]. Using porotic titanium scaffolds facilitates integration at the bone–scaffold interface and throughout the scaffold [5]. This is because the 3D printing process allows the precise customization of porous lattices, which induce bone ingrowth and ongrowth [14,15]. Greater osseointegration means long-term stability and faster mobilization. Most commonly, the material used is medical-grade titanium alloy powder (Ti-6Al-4V). It is the material of choice for load-bearing applications due to its high mechanical strength, low density, and good biocompatibility [16]. A significant concern with orthopedic implants is stress shielding, resulting in the remodeling, resorption, or loss of bone density (osteopenia), eventually leading to implant loosening and peri-implant fractures. Designing a 3D-printed titanium implant with controlled porosity reduces the elastic modulus and makes it more like natural bone. Therefore, porous implant design allows for homogenous stress distribution and maintenance of bone density around the implant [15,17]. Before insertion, the implant may be filled with cancellous autograft with the possible addition of stem cell allograft and bone marrow aspirate to improve osseointegration and bone regeneration [16,18]. Customized 3D-printed titanium implants are expensive and, as with any implant, there are potential complications to consider, including infection, hardware failure, and aseptic and septic non-union [19,20]. In their retrospective study on 39 patients treated with custom-made 3D-printed titanium implants for CSDs in the foot and ankle, Abar et al. reported that 25.6 of the implants had to be removed due to either septic or aseptic non-union within 22 months [17]. The technique is relatively new, and no long-term outcomes are reported in the literature. There are few articles published reporting on patients treated with custom-made titanium implants for diaphyseal and distal tibial bone defects and non-unions. This systematic review analyzes the results achieved with this technique to date.

## 2. Materials and Methods

### 2.1. Search Strategy

A systematic review of the medical literature was conducted in accordance with the Preferred Reporting Items for Systematic Reviews and Meta-Analyses (PRISMA) guidelines [8]. An electronic search of the PubMed, Scopus, and Cochrane databases was carried out. On PubMed, the following search string was used: ((((((“culture”[MeSH Terms] OR “culture”[All Fields] OR “custom”[All Fields] OR “customs”[All Fields] OR “customer”[All Fields] OR “customer s”[All Fields] OR “customers”[All Fields] OR “customization”[All Fields] OR “customizations”[All Fields] OR “customize”[All Fields] OR “customized”[All Fields] OR “customizes”[All Fields] OR “customizing”[All Fields]) AND (“tibia”[MeSH Terms] OR “tibia”[All Fields] OR “tibias”[All Fields] OR “tibia s”[All Fields] OR “tibiae”[All Fields])) OR (“titanium”[MeSH Terms] OR “titanium”[All Fields] OR “titanium s”[All Fields] OR “titaniums”[All Fields])) AND (“tibia”[MeSH Terms] OR “tibia”[All Fields] OR “tibias”[All Fields] OR “tibia s”[All Fields] OR “tibiae”[All Fields])) OR “TiAl6V4”[All Fields]) AND (“tibia”[MeSH Terms] OR “tibia”[All Fields] OR “tibias”[All Fields] OR “tibia s”[All Fields] OR “tibiae”[All Fields])) AND (English[Filter]). Scopus and Cochrane were searched with the following string: (((((custom) AND (tibia)) OR (titanium)) AND (tibia)) OR (TiAl6V4)) AND (tibia). To our knowledge, the first reported case of a custom-made 3D-printed titanium implant being utilized for the treatment of persistent non-union in the tibia dates to 2015, and the first reported case of bone loss in the tibia that was treated by such an implant was reported in 2016 [21,22]. The literature search covered the period from January 2015 to November 2024. The selection process for articles involved two stages. Initially, a screening process was implemented in which titles and abstracts were evaluated for their relevance. Subsequently, full-text articles deemed relevant were retrieved and evaluated for eligibility. Two reviewers (V.D.S. and G.M.) independently screened the abstracts and assessed the full articles. Any uncertainties or disagreements about including an article were resolved with the third reviewer (B.Z.).

### 2.2. Inclusion and Exclusion Criteria

The inclusion criteria were established following the “Population, Intervention, Comparison, Outcome, Study Design (PICOS)” framework. To qualify, studies needed to meet the following criteria. Population: cases or cohorts with patients aged 16 years or older suffering from segmental tibial defects or non-unions, which were reported in studies published between 2015 and 2024. Intervention: custom-made 3D-printed titanium implants used for treating segmental tibial defects or non-unions. Comparison: patients treated with custom-made 3D-printed titanium implants for segmental tibial defects or non-unions. Outcome: the primary outcome of interest was the osteointegration rate. The secondary outcomes included functional union, infection rate, the type and rate of other complications, and clinical and radiological outcomes. A minimum follow-up of at least one year was required for inclusion. Study design: Both experimental (randomized control trials—RCTs) and observational studies (prospective or retrospective cohort studies, case series, and case reports) published in English were included. Exclusion criteria were as follows: (1) studies on segmental bone defects resulting from the excision of tumors; (2) studies on segmental bone defects or non-unions involving the tibial plateau; (3) studies on post-traumatic segmental defects or non-unions in bones other than the tibia; (4) studies with inadequate follow-up; (5) studies on patients <16 years old; and (6) animal or biomechanical studies.

### 2.3. Data Extraction

The parameters analyzed included the number of patients, demographic information (age and sex), bone defect size, the osteointegration rate, the mean time to osteointegration, the functional union rate, the average follow-up duration, and the type and frequency of intra- and post-operative complications. Osteointegration was assessed radiographically, either with an X-ray or CT scan. In contrast, functional union was the subjective observation of the absence of pain and the ability to bear weight with the physiological load.

### 2.4. Quality Assessment

Two reviewers (V.D.S. and G.M.) evaluated the quality of the included studies using the Methodological Index for Non-Randomized Studies (MINORS) score [23]. The MINORS consists of 12 items, with the first 8 applying specifically to non-comparative studies. The maximum score for non-comparative studies is 16, while for comparative studies, it is 24.

## 3. Results

**Figure 1:**
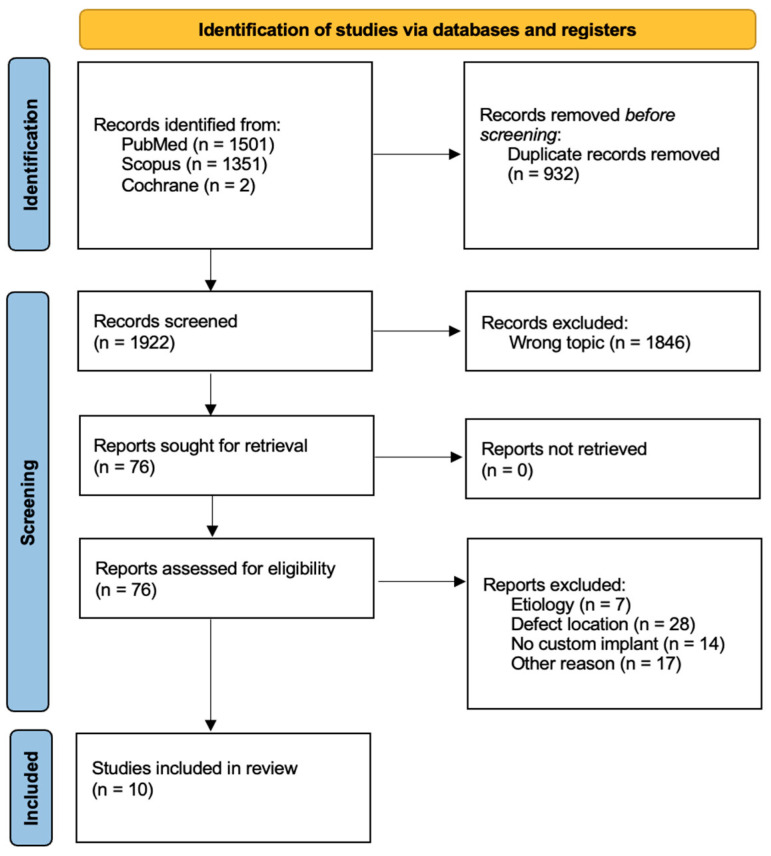
PRISMA flow chart of literature search.

A total of 2854 studies were initially identified through the literature search. After screening, 76 studies were retrieved and assessed for eligibility. Ten of these studies met the inclusion criteria (Figure 1) and were subsequently evaluated for methodological quality according to the MINORS criteria (Table 1). The scores ranged from 8 [18,21,22,24,25] to 12 [26], with an average score of 9, indicating an overall moderate methodological quality (CR, case report; PS, prospective study; and RS, retrospective study). The descriptive characteristics of the included studies are presented in Table 2. A total of 19 patients (13 male and 6 female) were included, with a mean age of 43.1 years (range of 25 to 65 years) [25,26]. Eight patients reported a tibial diaphyseal defect, and eleven of them reported a tibial distal defect. The included studies reported data on a single patient in seven out of ten cases. The remaining three studies included between two and six patients each [26,27,28]. Osteomyelitis following trauma, either due to an open fracture or severe soft tissue damage, was recorded in ten cases [26,27,28,29], while non-union was detected in six patients (five aseptic [12,24,26,30] and one septic non-union [25]); severe bone loss after trauma occurred in three cases [16,18,27]. In the majority of cases, either autografts or allografts were utilized [16,18,22,25]. Gamieldien et al. [29] employed bone marrow aspirate (RIA—reamer–irrigator–aspirator) to enhance osseointegration and bone regeneration. In two studies, the use of bone grafts was not specified [28,30]. Beatti et al., Hou et al. and Liu et al. [24,26,27] did not use any grafts. The follow-up duration ranged from 12 to 45 months [28,29], averaging 19.2 months. The outcomes of the included studies are presented in Table 3. The mean size of the bone defect was reported in four studies and varied from 3 cm [29] to 8.5 cm [22,24]. In the studies where it was reported, osteointegration was consistent in all patients. Indeed, one study did not specify the occurrence of osteointegration [25]. The time to osteointegration was reported in four studies [18,21,27,28], ranging from 4 [28] to 6 [18] months with a mean of 5.3 months. Functional union was achieved in all cases except for one (94.7%) [26]. The average time to functional union was reported in eight studies (all except Beatti et al. [27] and Caravelli et al. [28]), ranging from 0.4 to 4.1 months with a mean of 2.1 months. The complications that arose are listed in Table 4. Three complications were reported among the 19 patients treated, resulting in a 16% complication rate. All of these occurred in patients previously affected by osteomyelitis. Specifically, there was one wound-healing delay, one nail removal due to intolerance, and one angular deformity greater than 5° [26,28].

## 4. Discussion

This systematic review aims to analyze the results achieved with customized 3D-printed titanium implants for tibial bone defects and non-unions, evaluating outcomes and potential determinants of complications and failure. The most common traditional methods for treating bone defects and non-unions include massive bone grafting, induced membrane techniques, and distraction osteogenesis. In some cases, amputation is necessary [31]. Recently, customized 3D-printed implants have been employed to address CSDs and non-unions, especially when traditional interventions fail. The effectiveness of established techniques, such as DO and Masquelet, has been proven over time. However, these methods are often associated with extended treatment durations and the need for multiple surgeries, which require patient compliance [10]. Customized 3D-printed porous titanium implants represent a promising solution, as previously mentioned, because they offer several advantages. First, they are tailored to the patient’s needs, ensuring an exact fit for the bone defect. Second, compared to bone allografts, they can deliver biological agents, exhibit greater strength, and lower the risk of immune rejection [17]. Additionally, the manufacturing process allows for precise control over porosity, which helps mitigate the risks of stress shielding and bone resorption. These features should lead to a single operation with reduced operating time, aiming to facilitate rapid mobilization [26,29,32,33,34]. However, a combination of the induced membrane technique and custom-made implantation has been reported to yield good outcomes in terms of stability, painless weight bearing, and return to daily activities [29,30]. It is also important to note that factors beyond stress shielding influence implant longevity. For example, micromotion between the implant and the bone can hinder bony ingrowth, and excessive micromotion may lead to implant loosening or breakage. In this context, titanium has shown favorable outcomes due to its lightweight strength and ability for osseointegration [21]. We performed a systematic review, reporting 19 cases of patients who underwent this technique after either non-union, infection, or severe bone loss following trauma. Despite the lack of univocal and standard score evaluation, good results were reported even in segmental defects measuring 8.5 cm [22,24]. Osteointegration was specified in all studies except for one and it was observed within 6 months in all patients. Functional union was achieved in 94.7% of the cases. It should be noted that traditional techniques can take as long as custom-made implants to achieve bone union, if not longer. In addition, customized 3D-printed porous titanium implants seem to allow faster mobilization due to an immediate stable construction. They are better tolerated by the patient, especially compared to external fixation and DO, with no risk of donor graft site morbidity [31,35]. However, a 16% complication rate has been reported, all in patients with osteomyelitis, suggesting that infection is one of the most concerning prognostic factors. Moreover, Abar et al. [17] reported that approximately 25% of patients treated with 3D-printed implants in open wounds required removal due to non-union, a rate comparable to existing fixation methods such as DO and Masquelet [4]. Indeed, this treatment option is not without its disadvantages. It is a costly process. However, some studies have indicated that 3D customized implants may offer a cost-effective surgical option for patients at high risk of nonunion. Furthermore, studies have revealed that the lifetime costs of amputation are significantly higher than limb salvage [16,20,24]. Nevertheless, as this technology continuously improves, it is reasonable to posit that its use will become more widespread, facilitating greater economic access. Another critical factor to consider is the time required for the design of each implant, for which technological improvements will be necessary [18,36]. A further potential disadvantage is the risk of contamination leading to infection. However, this risk is inherent to any implant, especially in case of pre-existing infection, such as in the cases reported in this review [18]. Comparing this innovative approach with traditional techniques, Hsu et al. reported an infection rate of 27.3% in patients treated with the induced membrane technique, and Wakefield et al. observed a union rate of 94.6% and 88.0% in patients treated with DO and Masquelet, respectively [9,12]. It is, therefore, evident that the results obtained provide a promising foundation upon which to build. This finding indicates that the proposed solution may be a valid approach for the standard management of segmental defects and non-unions in the future. The present study is subject to several limitations. Firstly, a low number of studies and, consequently, patients were included. Our results summarized the outcomes reported in the existing literature. Still, there were not enough data to draw meaningful conclusions or create a new standard of care with explicit guidelines on treatment using custom-made 3D-printed titanium implants. Furthermore, the overall methodological quality of the included studies was only moderate. Most included studies were case reports with no uniform reporting system for bone and functional results. The time to osteointegration and the size of the bone defect were only reported in a minority of studies. There was only one prospective study, and no randomized controlled trials reported results obtained with custom-made 3D-printed titanium implants for the treatment of segmental tibial defects or non-unions. In addition, it is imperative to acknowledge that, as this is a novel and pioneering treatment, authors are inclined to present cohorts with predominantly favorable outcomes. This engenders awareness within the orthopedic community regarding complications whilst obscuring the actual rate of complications. Finally, detailed information on individual patient status and comorbidities was not sufficiently reported, making it impossible to include these factors in this systemic review to find possible prognostic determinants. Finally, innovative approaches, such as customized implants, may pave the way for new treatments that patients better tolerate and allow early mobilization. Conversely, it is important to highlight that this new technique is not without its challenges and further studies are necessary to fully assess its role compared to traditional techniques. The current literature does not provide clear treatment guidelines, but the potential for custom-made 3D-printed titanium implants in this field is promising. Further studies, particularly by defect location and using a universal scoring system, are needed to evaluate the potential advantages of customized 3D-printed porous titanium implants over traditional techniques, draw new treatment guidelines, and assess the biomechanical implications of these implants to reduce the risk of complications such as stress shielding and implant loosening.

## 5. Conclusions

Custom-made 3D-printed titanium implants constitute a novel and pioneering treatment modality for managing segmental tibial defects and non-unions. This treatment approach attains bone union rates comparable to those observed with conventional techniques, such as DO and Masquelet. Consequently, both treatment modalities represent valid options for addressing these orthopedic challenges. Additional studies are required to assess the potential benefits of customized 3D-printed porous titanium implants over traditional techniques.

## Figures and Tables

**Table 1:** MINORS scores of included studies.

Study	Year	Design	MINORS
Beatti et al. [27]	2022	RS	10
Brown et al. [18]	2022	CR	8
Caravelli et al. [28]	2022	RS	10
Gamieldien et al. [29]	2023	RS	10
Hamid et al. [22]	2016	CR	8
Hou et al. [24]	2022	CR	8
Hsu et al. [21]	2015	CR	8
Kachare et al. [30]	2024	CR	8
Liu et al. [26]	2022	PS	12
Tang et al. [25]	2023	CR	8

CR, case report; PS, prospective study; and RS, retrospective study.

**Table 2:** Descriptive characteristics of included studies.

Author/s	Year	Treatment Period	Patients	Mean Age (Range)	M/F Ratio	Defect Location	Mean Follow-Up (Range) (Months)
Beatti et al. [27]	2022	2016–2019	2	47 (40–54)	1/1	1 tibial diaphysis, 1 distal tibia	N.A. (12–N.A.)
Brown et al. [18]	2022	N.A.	1	26 (N.A.)	1/0	Distal tibia	36 (N.A.)
Caravelli et al. [28]	2022	2016–2020	4	59.3 (55–64)	3/1	4 distal tibia	29.5 (18–45)
Gamieldien et al. [29]	2023	2019–2022	1	37 (N.A.)	1/0	Distal tibia	12 (N.A.)
Hamid et al. [22]	2016	N.A.	1	46 (N.A.)	0/1	Distal tibia	13 (N.A.)
Hou et al. [24]	2022	N.A.	1	42 (N.A.)	0/1	Tibial diaphysis	26 (N.A.)
Hsu et al. [21]	2015	N.A.	1	63 (N.A.)	1/0	Distal tibia	12 (N.A.)
Kachare et al. [30]	2024	N.A.	1	38 (N.A.)	1/0	Distal tibia	18 (N.A.)
Liu et al. [26]	2022	2017–2022	6	47.2 (32–65)	5/1	5 tibial diaphysis, 1 distal tibia	21.5 (13–35)
Tang et al. [25]	2023	N.A.	1	25 (N.A.)	0/1	Tibial diaphysis	12 (N.A.)

N.A., not available; M/F, male/female.

**Table 3:** Outcomes of included studies.

Author/s	Year	Mean Bone Defect Size (Range) (cm)	Osteointegration Rate (%)	Time to Osteointegration (Range) (Months)	Functional Union Rate (%)	Time to Functional Union (Range) (Months)
Beatti et al. [27]	2022	N.A.	100%	4.9 (N.A.)	100%	N.A. (1.5–1.6)
Brown et al. [18]	2022	N.A.	100%	6 (N.A.)	100%	1.4 (N.A.)
Caravelli et al. [28]	2022	N.A.	100%	N.A. (4–6)	100%	N.A.
Gamieldien et al. [29]	2023	3 (N.A.)	N.A.	N.A.	100%	2.6 (N.A.)
Hamid et al. [22]	2016	8.5 (N.A.)	100%	N.A.	100%	4.1 (N.A.)
Hou et al. [24]	2022	8.5 (N.A.)	100%	N.A.	100%	1 (N.A.)
Hsu et al. [21]	2015	N.A.	100%	5 (N.A.)	100%	3 (N.A.)
Kachare et al. [30]	2024	N.A.	100%	N.A.	100%	3 (N.A.)
Liu et al. [26]	2022	N.A. (6–N.A.)	100%	N.A.	83.3%	0.4 (0.2–0.5)
Tang et al. [25]	2023	5 (N.A.)	N.A.	N.A.	100%	1 (N.A.)

N.A., not available.

**Table 4:** Complications.

Author/s	Pts	DU or NU	AD > 5°	Deep, Persistent, or Recurrent Infection	IL or Breakage	LLD > 2.5 cm	Joint Related Complication	Other Complications	Complications per Patient
Beatti et al. [27]	2	0	0	0	0	0	0	0	0 (0/2)
Brown et al. [18]	1	0	0	0	0	0	0	0	0 (0/1)
Caravelli et al. [28]	4	0	0	0	0	0	0	1 wound-healing delay 1 nail removal due to intolerance	0.5 (2/4)
Gamieldien et al. [29]	1	0	0	0	0	0	0	0	0 (0/1)
Hamid et al. [22]	1	0	0	0	0	0	0	0	0 (0/1)
Hou et al. [24]	1	0	0	0	0	N.A.	N.A.	N.A.	0 (0/1)
Hsu et al. [21]	1	0	0	0	0	0	0	0	0 (0/1)
Kachare et al. [30]	1	0	N.A.	N.A.	0	N.A.	N.A.	N.A.	0 (0/1)
Liu et al. [26]	6	0	1	0	0	0	N.A.	N.A.	0.2 (1/6)
Tang et al. [25]	1	0	0	0	0	0	0	N.A.	0 (0/1)

AD, angular deformity; DU, delayed union; IL, implant loosening; LLD, leg length discrepancy; N.A., not available; NU, non-union.

## Data Availability

The datasets generated during and/or analyzed during the current study are available from the corresponding author upon reasonable request.

## Abbreviations

The following abbreviations are used in this manuscript:

DO	Distraction osteogenesis
IMT	Induced membrane technique
CSD	Critically sized defect
